# PD141 Health Technology Assessment Methodology Approach For Precision Personalized Medicine: An Innovative Public Procurement Case Study

**DOI:** 10.1017/S0266462324003775

**Published:** 2025-01-07

**Authors:** Agnieszka Dobrzynska, Patricia Julia Garcia-Sanz, María Piedad Rosario Lozano, Lorena Aguilera-Cobos, Juan Antonio Blasco-Amaro

## Abstract

**Introduction:**

Innovative public procurement (IPP) is a driver of innovation across sectors. IPP involves the strategic acquisition of cutting-edge technologies and solutions by public entities in collaboration with the private sector. This approach aims to leverage the potential of “omics” technologies, such as genomics and proteomics, within the public sector to advance healthcare solutions.

**Methods:**

The IPP process comprises key phases such as needs identification, solicitation preparation, execution, evaluation and awarding, and impact and assessment. The methodology applied was based on the Rapid Assessment Tool for Omics Technologies developed by the Andalusian Agency for Health Technology Assessment, accompanied by clinical validity reports from the impact and evaluation phase of the Technical Office for IPP of Andalusia. The aim was to assess the clinical validation results of two omics technologies, that is, two diagnostic tests. A systematic review was performed to identify existing evidence. We also addressed the challenges associated with implementing the Rapid Assessment Tool in the IPP process.

**Results:**

Systematic reviews identifying evidence on the clinical validity and utility of omics technologies provided a foundation for the subsequent evaluation of two technologies in development, once the clinical trials had finished. An analysis of scientific evidence, together with the compilation of information provided by industry, was conducted through a questionnaire and clinical data derived from the company’s studies. The analysis of clinical and diagnostic validity was not conclusive. We delivered the final assessment report to support decision-making in the public health system of Andalusia.

**Conclusions:**

Both assessed technologies presented a high degree of innovation, but different challenges and issues were identified during the application of the Rapid Assessment Tool for Omics Technologies. Further improvement in IPP procedures for innovative technologies in Andalusia, including integration of our methodological approach at the start the IPP process, could facilitate the acquisition of cutting-edge technologies in collaboration with public entities.

